# Spatiotemporal characterization of breathing-induced B_0_ field fluctuations in the cervical spinal cord at 7T

**DOI:** 10.1016/j.neuroimage.2017.11.031

**Published:** 2018-02-15

**Authors:** S. Johanna Vannesjo, Karla L. Miller, Stuart Clare, Irene Tracey

**Affiliations:** Wellcome Centre for Integrative Neuroimaging, FMRIB, Nuffield Department of Clinical Neurosciences, University of Oxford, Oxford, UK

**Keywords:** MRI, Spinal cord, 7T, Physiological noise, B_0_ field fluctuations, Breathing, BMI, Body Mass Index, EPI, Echo-Planar Imaging, FLASH, Fast Low Angle Shot, MRI, Magnetic Resonance Imaging, PCA, Principal Component Analysis, PC, Principal Component

## Abstract

Magnetic resonance imaging and spectroscopy of the spinal cord stand to benefit greatly from the increased signal-to-noise ratio of ultra-high field. However, ultra-high field also poses considerable technical challenges, especially related to static and dynamic B_0_ fields. Breathing causes the field to fluctuate with the respiratory cycle, giving rise to artifacts such as ghosting and apparent motion in images. We here investigated the spatial and temporal characteristics of breathing-induced B_0_ fields in the cervical spinal cord at 7T.

We analyzed the magnitude and spatial profile of breathing-induced fields during breath-holds in an expired and inspired breathing state. We also measured the temporal field evolution during free breathing by acquiring a time series of fast phase images, and a principal component analysis was performed on the measured field evolution.

In all subjects, the field shift was largest around the vertebral level of C7 and lowest at the top of the spinal cord. At C7, we measured peak-to-peak field fluctuations of 36 Hz on average during normal free breathing; increasing to on average 113 Hz during deep breathing. The first principal component could explain more than 90% of the field variations along the foot-head axis inside the spinal cord in all subjects.

We further implemented a proof-of-principle shim correction, demonstrating the feasibility of using the shim system to compensate for the breathing-induced fields inside the spinal cord. Effective correction strategies will be crucial to unlock the full potential of ultra-high field for spinal cord imaging.

## Highlights

• The B_0_ field in the spinal cord fluctuates with the breathing cycle.
• Average peak-to-peak ΔB_0_ of 36/113 Hz at C7 during normal/deep breathing at 7T.
• The first principal component explains more than 90% of the field variance.
• Respiratory trace correlates well with field fluctuations during normal breathing.
• Proof-of-principle correction using 2nd-order shims was demonstrated.

## Introduction

The spinal cord is a challenging structure to image with MRI, due to its thin elongated shape, its location deep inside the body and nearby physiological influences. At its widest, in the cervical section of the spine, it only measures about 1 cm in diameter. Because of the small cross-sectional dimensions, high-resolution imaging is necessary to resolve substructures within the spinal cord, such as gray/white matter distribution and parcellation. Consequently, imaging at higher background field strength should be advantageous, as the benefit in signal-to-noise ratio can be translated into higher image resolution. However, despite the potential advantage, ultra-high field spinal cord imaging has been slow to develop. Partly, this is because the increased background field also exacerbates a number of technical challenges of spinal cord imaging, particularly concerning B_0_ and B_1_ field distributions. Optimized transmit and receive B_1_ fields through improved coil design for 7T spinal cord imaging has been the subject of a number of publications in recent years (Sigmund et al., 2012, Vossen et al., 2011, Wu et al., 2009; Zhang et al., 2017, Zhao et al., 2014). In contrast, B_0_ field imperfections have received less attention, and remain among the most pressing obstacles to harnessing the full potential of ultra-high field for spinal cord imaging. While both static and dynamic B_0_ field imperfections lead to significant image artifacts, we will mainly focus on dynamic field instabilities in this work.

The background B_0_ field distribution is distorted by differences in magnetic susceptibility, especially between air and tissue (Schenck, 1996). As large air-filled cavities, the lungs are a major factor contributing to shape the field distribution in surrounding regions, including the spinal cord. Breathing is associated with changing lung volume and motion of the chest wall, which in turn causes the B_0_ field to vary periodically with the respiratory cycle, even at some distance from the chest (Raj et al., 2000, Raj et al., 2001, Van de Moortele et al., 2002). In the brain, at 7T, breathing-induced field shifts of about 1–4 Hz have been measured during normal breathing in healthy volunteers (Duerst et al., 2016, Van de Moortele et al., 2002, van Gelderen et al., 2007), giving rise to a variety of artifacts. These include ghosting, blurring and signal intensity modulation in anatomical T2*-weighted acquisitions (Duerst et al., 2016, van Gelderen et al., 2007, Versluis et al., 2010), apparent motion in EPI time-series (Raj et al., 2001, Van de Moortele et al., 2002, van Gelderen et al., 2007) and line-broadening in spectroscopy (Wilm et al., 2014).

Due to the proximity of the lungs to the spine, breathing-induced field fluctuations would be expected to have an even larger impact on spinal cord imaging. One study at 3T has reported breathing-induced field shifts, as measured by field maps acquired during expired and inspired breath-holds, of about 70 Hz in the cervical spinal cord at the vertebral level of C7 (Verma and Cohen-Adad, 2014). The effect should increase with background field strength; however the situation in the spinal cord at ultra-high field remains to be investigated. Moreover, breath-holds are not entirely representative of normal conditions during image acquisition, and do not give information about the field variations over the full breathing cycle. The temporal component of the breathing-induced field fluctuations will be especially important to understand when it comes to designing suitable correction strategies to counteract the effects of the field fluctuations.

In this work, we investigate breathing-induced B_0_ field fluctuations in the cervical spinal cord at 7T. We study the spatial field distribution in field maps acquired during breath-holds, similar to the study reported by Verma et al. Furthermore, we acquire fast phase-sensitive gradient-echo images during free breathing, in order to temporally resolve the changing B_0_ field over the full breathing cycle. The temporal and spatial characteristics of the breathing-induced fields are then analyzed with a view to potential correction strategies.

## Methods

All measurements were performed on a Siemens Magnetom whole-body 7T system (Siemens Healthineers, Erlangen, Germany), using a volume-transmit, 16-channel receive cervical spine coil (Quality Electrodynamics, Mayfield Village, OH, USA). In vivo scanning was performed in compliance with local ethics guidelines.

### Breath-hold field characterization

In a first set of experiments, we characterized the magnitude and spatial profile of the breathing-induced field shifts in field maps acquired during breath-holds, similarly to the 3T study reported by Verma et al. Sagittal field maps (11 slices, FOV = 152 × 152 mm^2^, 2 × 2 × 2 mm^3^ resolution, FA = 20°, TR = 80 ms, TE1 = 4.08 ms, TE2 = 5.1 ms, bandwidth 506 Hz/pixel, acquisition time 12 s) were acquired in nine healthy volunteers (6 male, mean (range) height 1.76 (1.63–1.85) m, weight 71 (49–88) kg, BMI 22.9 (18.4–27.8) kg/m^2^, age 31 (23–43) years). Anatomical coverage in the foot-head direction varied slightly between subjects, but always included the full cervical spinal cord. The acquisitions were performed during breath-holds in an either expired or inspired breathing state, and were repeated 2–3 times per condition in each subject. Before each breath-hold, the subjects were given a breathing pace to follow (‘breathe in, breathe out, hold’ or ‘breathe in, breathe out, breathe in, hold’) in order to synchronize the start of the breath-hold with the start of the acquisition.

For each pair of successively acquired expired and inspired field maps, the field difference, $\text{Δ}B_{0}\left(r\right)$, was calculated as:

(1) $$\text{Δ}B_{0}\left(r\right)=\frac{1}{\gamma }\cdot \frac{arg\left(\frac{e^{i\varphi_{in}\left(r\right)}}{e^{i\varphi_{ex}\left(r\right)}}\right)}{\text{Δ}TE},$$

where $\varphi_{ex}\left(r\right)$ and $\varphi_{in}\left(r\right)$ denote the expired and inspired field maps scaled to radians. The original field maps contain a phase contribution both from static susceptibility-induced field offsets and the respiratory-induced field:

$$\varphi_{in}=\varphi_{static}+\text{Δ}\varphi_{in}$$

$$\varphi_{out}=\varphi_{static}+\text{Δ}\varphi_{out}\text{.}$$

The complex division yields the phase difference, $\text{Δ}\varphi_{in}-\text{Δ}\varphi_{out}$,

$$\frac{e^{i\varphi_{in}\left(r\right)}}{e^{i\varphi_{ex}\left(r\right)}}=e^{i\left(\varphi_{in}\left(r\right)-\varphi_{out}\left(r\right)\right)}=e^{i\left(\text{Δ}\varphi_{in}\left(r\right)-\text{Δ}\varphi_{out}\left(r\right)\right)}$$

and is therefore insensitive to phase wraps in $\varphi_{ex}\left(r\right)$ and $\varphi_{in}\left(r\right)$ caused by the static field component, $\varphi_{static}$. Hence no spatial unwrapping of $\varphi_{ex}\left(r\right)$ and $\varphi_{in}\left(r\right)$ was performed. The calculated $\text{Δ}B_{0}\left(r\right)$ is free from wraps provided that $\gamma \text{Δ}B_{0}\left(r\right)\text{Δ}TE<\pi ,\;\forall \left(r\right)$. With a ΔTE of about 1 ms this corresponds to a $\text{Δ}B_{0}$ of up to 500 Hz. No spatial registration between the expired and the inspired field maps was performed.

For each subject, a mask covering the spinal cord and lower brainstem was manually defined in the magnitude image of the field map acquisitions. The calculated $\text{Δ}B_{0}\left(r\right)$ was then averaged over all voxels inside the spinal cord mask in the transverse plane, yielding a one-dimensional measure of the respiratory induced field offset, $\text{Δ}B_{0}\left(z\right)$, along the foot-head direction. The measured $\text{Δ}B_{0}\left(z\right)$ was subsequently averaged over all trials for each subject. In order to compare results between subjects, a shared foot-head axis was constructed by defining the top of the C1 and bottom of the C7 vertebrae as reference points and linearly scaling each individual z-axis accordingly. The measured field offsets were subsequently averaged over all subjects.

The following mathematical model, suggested by Verma et al., was fit to the measured field offsets:

(2) $$y=\frac{a}{\sigma \sqrt{2\pi }}e^{-\frac{{\left(z-\mu \right)}^{2}}{2\sigma^{2}}}\left(1+erf\left(\frac{s(z-\mu )}{\sqrt{2}}\right)\right)+bz+c.$$

The model is comprised of a linear term with slope *b*, and a skewed Gaussian function, where *a* is a scaling factor, *μ* and *σ* are the mean and standard deviation, *erf* represents the error function and *s* is a skewness parameter. A constant, *c*, was added to the model to account for axis shifts. The z-axis was scaled such that the top of C1 corresponded to 0 and the bottom of C7 to 1. The six parameters *a*, *μ, σ*, *s*, *b* and *c* were first fit to the mean field offset based on a set of heuristically selected starting points. The resulting fit parameters were then used as starting point in fitting the model to the measured field offset for each individual subject.

### Free-breathing field characterization

The breath-hold field map acquisitions yield a spatial characterization of the breathing-induced field offsets, but no temporal information. In order to resolve the respiratory field variations over time, a time-series of fast gradient-echo images (FLASH – Fast Low Angle SHot) (Haase et al., 1986) were acquired. The phase images contain a contribution from the B_1_ transmit/receive phase and the B_0_ field offset at the time of acquisition. Assuming that the B_1_-induced phase is static (as assumed in standard field mapping), the phase difference between images in the time-series reflect the change in B_0_ field between the acquisition time points. Removing the static component from the time-series of phase images thus removes both the B_1_-induced phase and the static susceptibility-induced B_0_ field offset, leaving only the time-varying B_0_ field component. A similar approach using EPI acquisitions has previously been employed to measure breathing-induced field fluctuations in the brain at 7T (van Gelderen et al., 2007). In this work, we chose FLASH acquisitions rather than EPI to avoid severe image distortion and signal drop-out caused by the large static B_0_ field offsets in the spine at 7T.

FLASH acquisitions of a single sagittal slice were acquired with the following parameters: FOV = 144 × 144 mm^2^, 3.4 × 2.3 × 3.0 mm^3^ resolution, FA = 6°, TR = 8 ms, TE = 4.08 ms, bandwidth 240 Hz/pixel, volume TR 344 ms, 200–400 repetitions (∼1–2 min T_acq_). The sequence parameters were chosen such as to yield sufficient field-of-view to avoid aliasing into the spinal cord and sufficient resolution to have at least two-three voxels inside the spinal cord in each transversal plane, while minimizing the volume TR, which governs the temporal resolution. A volume TR of 344 ms means that 12 samples are obtained in a normal breathing cycle of about 4 s. This should be enough to resolve the breathing-induced fields over time with reasonable accuracy.

The FLASH acquisitions were obtained during free breathing in ten healthy volunteers (7 male, mean (range) height 1.77 (1.63–1.88) m, weight 72 (52–93) kg, BMI 22.7 (19.6–27.2) kg/m^2^, age 32 (23–43) years). The acquisitions were repeated three times in each subject, and the subjects were instructed in the different acquisitions to either breathe normally, breathe deeply or perform expired/inspired breath-holds on cue. During all acquisitions, a trace from a respiratory bellows was simultaneously recorded. The bellows (76513NM10, Lafayette Instruments, USA) was attached to a gauge pressure sensor (24PCEFA6G, Honeywell, USA) connected to a transducer amplifier (DA100C, Biopac Systems Inc, USA), and the signal was recorded via a Biopac acquisition system. In six of the subjects the receive chain for the respiratory trace included a high-pass filter, and in four of the subjects the filter was turned off.

The phase in each voxel, φ(r,t), was unwrapped over time and the mean phase over the time-course, $\bar{\varphi }\left(r\right)$, was calculated. The field fluctuation time-course, $\text{Δ}B_{0}\left(r,t\right)$, was then calculated as:

(3) $$\text{Δ}B_{0}\left(r,t\right)=\frac{1}{\gamma }\cdot \frac{\varphi \left(r,t\right)-\bar{\varphi }\left(r\right)}{TE}\text{.}$$

For breath-hold acquisitions, $\bar{\varphi }\left(r\right)$ was obtained from a stretch of normal free breathing before each breath-hold. No spatial registration of the image time-series was performed. A mask covering the spinal cord was manually defined on the mean of the magnitude images. At each time point, the average of $\text{Δ}B_{0}\left(r,t\right)$ inside the spinal cord mask in the transverse plane was calculated, yielding the field offset as a function of foot-head position and time, $\text{Δ}B_{0}\left(z,t\right)$. The z-axis was normalized between subjects by taking C1 and C7 as reference points, as described above.

### Spatiotemporal analysis

For the sagittal FLASH acquisitions, a principal component analysis (PCA) was performed on 30 s of the measured $\text{Δ}B_{0}\left(z,t\right)$ for each breathing condition – normal, deep and breath-hold. In the case of breath-holds, only time points within the breath-holds were included in the PCA. Occasional swallowing events were excluded from the analysis in all cases. The PCA yielded a set of spatial principal components (PCs, corresponding to the z direction) and associated projection time-courses, in order of variance explained.

To estimate the peak-to-peak magnitude of the field fluctuations induced by the different breathing conditions, the difference between the maximum and the minimum value of the time-course of the first PC was evaluated. The estimate was based on the PC analysis, instead of the directly measured field time-courses, as a means of de-noising. The peak-to-peak values are representative of the range of fields encountered during the different breathing conditions, and are directly comparable to the breath-hold field map data.

In order to evaluate the feasibility of using the respiratory trace as an external tracker to yield an estimate of the breathing-induced fields, $\text{Δ}{\hat{B}}_{0}\left(z,t\right)$, a separable linear model was assumed:

(4) $$\text{Δ}{\hat{B}}_{0}\left(z,t\right)=\text{Δ}B_{0,ref}\left(z\right)\cdot R\left(t\right)\text{,}$$

where *R(t)* is the signal from the respiratory bellows. 30 s of normal breathing was used as training data to determine $\text{Δ}B_{0,ref}\left(z\right)$, which was subsequently used to predict the expected field time-course for all breathing conditions. The size of residuals ($\Delta B_{0}\left(z,t\right)-\Delta {\hat{B}}_{0}\left(z,t\right)$) was quantified by the temporal standard deviation at each spinal cord level.

In order to evaluate the potential predictive power of a linear model with a single temporal tracker in the ideal case, the principal component analysis was employed. The first PC of normal breathing was determined from 30 s of training data. Subsequent measured field time-courses of different breathing conditions were then projected onto the first PC of normal breathing, yielding time-courses of the field explained by that spatial component. Similarly to the respiratory trace model, the field not captured by the first PC was quantified by the temporal standard deviation of the residual.

### In-plane field characterization

The analysis up to this point considered only the measured field fluctuations inside the spinal cord itself, and averaged out any field gradients in the transverse plane. To explore the full in-plane spatial distribution of the breathing-induced field fluctuations, transversal FLASH images (FOV = 146 × 180 mm^2^, 3.5 × 2.8 × 3.0 mm^3^ resolution, FA = 6°, TR = 8 ms, TE = 4.08 ms, bandwidth 240 Hz/pixel, volume TR 336 ms) were acquired at vertebral levels C3, C5 and C7 during normal free breathing in two subjects. The field time-course of each voxel, $\text{Δ}B_{0}\left(r,t\right)$, was calculated as described above.

In-plane analyses were performed on the transversal and sagittal image time-series of normal breathing. A magnitude mask was created for each slice by applying a threshold to the mean magnitude images. Manual editing of the mask was subsequently performed to exclude voxels with phase instabilities, as manifested by unwrapping errors in the phase time-courses, φ(r,t). A principal component analysis of the field time-courses of the voxels within the magnitude mask was performed separately for each slice, yielding a set of spatial in-plane principal components, each associated with one projection time-course.

To simulate a correction based on the respiratory trace model, the $\text{Δ}B_{0,ref}\left(z\right)$ obtained from the sagittal acquisition was used together with the respiratory trace of each acquisition to predict $\text{Δ}{\hat{B}}_{0}\left(z,t\right)$, which was then subtracted from the measured time-courses. Additionally, the potential for field compensation using the shim system was evaluated by removing the in-plane spatial contributions of up to 1st, 2nd or 3rd-order spherical harmonics. Finally, the residual after subtracting the contribution from the first in-plane principal component was evaluated for comparison. Retrospective correction of field fluctuations of arbitrary in-plane spatial profile may be possible at the level of image reconstruction, if the spatiotemporal structure is known. In each case, the residual field was quantified by the temporal standard deviation.

### Anatomical multi-echo GRE

In order to demonstrate the type of artifacts that can arise due to the breathing-induced field fluctuations, a high-resolution multi-echo GRE acquisition (19 transversal slices, FOV = 180 × 158 mm^2^, resolution 0.35 × 0.35 × 3.0 mm^3^, FA = 51°, TR = 700 ms, TE = 6.3/10.6/14.9/19.2/23.5/27.8/32.1 ms, bandwidth 260 Hz/pixel, T_acq_ 5:15 min) was obtained in one of the subjects. The subject was instructed to breathe normally during the acquisition. No phase stabilization was applied to the data.

### Shim correction

In one subject, a proof-of-principle shim correction was implemented to compensate for the breathing-induced fields. Initially, expired/inspired field maps were acquired with a fixed shim field setting. Up to 2nd-order spherical harmonic shim fields were then fitted to the field difference within the spinal cord mask, assuming expiration as reference state. The calculated correction shim settings were subsequently applied during the inspired condition of a new field map acquisition pair within the same scan session. The compensated acquisition pair was repeated twice.

## Results

### Breath-hold field maps

**Fig. 1:**
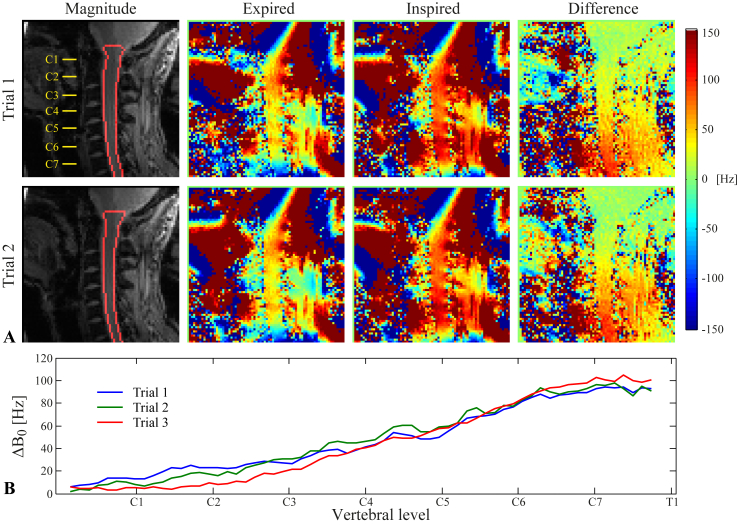
A) Two trials of the field map acquisition during expired/inspired breath-holds in one subject. Displayed are the magnitude image with an outline of the spinal cord mask (left), the expired/inspired field maps (center) and the difference between the field maps (right). B) The measured $\text{Δ}B_{0}\left(z\right)$ inside the spinal cord, shown for all three trials acquired in this subject.

Fig. 1 shows field maps acquired during breath-holds, together with the resulting $\text{Δ}B_{0}\left(z\right)$, for one subject. Difference images between expired and inspired breath-hold field maps show a systematic field difference between the two breathing states, appearing as a smooth, spatially varying field offset (Fig. 1A). The field offset was larger towards lower levels of the cervical spine. The calculated $\text{Δ}B_{0}\left(z\right)$ peaked at about 100 Hz around the vertebrae C7-T1, falling to about 5–10 Hz at C1 (Fig. 1B). The magnitude and spatial profile of $\text{Δ}B_{0}\left(z\right)$ was fairly reproducible between trials, with a variability of around 5–20 Hz.

**Fig. 2:**
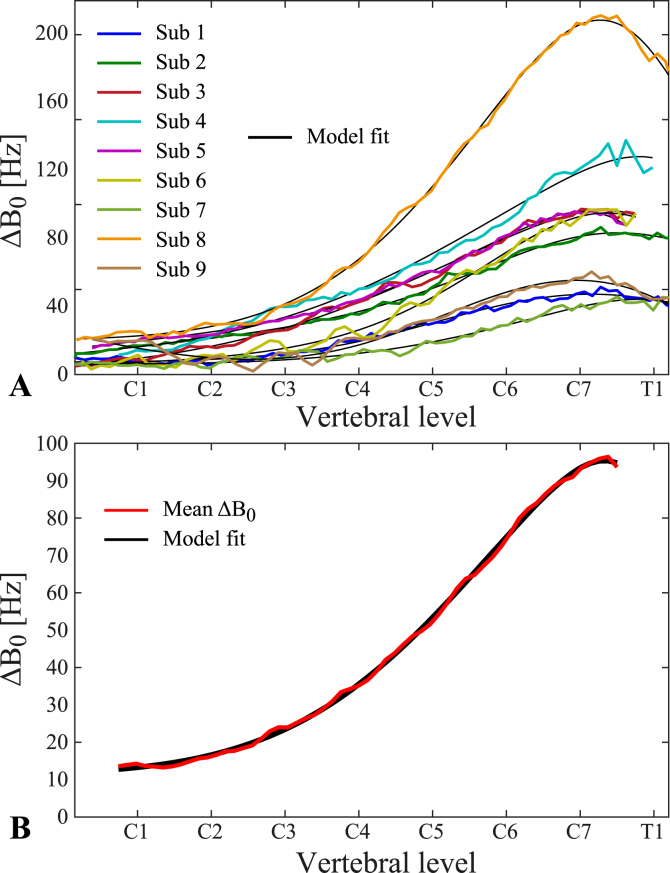
Breathing-induced field offsets as measured by the difference between expired/inspired breath-hold field map acquisitions. A) $\text{Δ}B_{0}\left(z\right)$ for each individual subject, averaged over all trials, overlaid on the fitted skewed Gaussian model. B) Average $\text{Δ}B_{0}\left(z\right)$ over all subjects, overlaid on the fitted skewed Gaussian model.

The measured $\text{Δ}B_{0}\left(z\right)$ for all subjects are shown in Fig. 2. In each subject, a peak around the vertebral levels of C7-T1 is observed, from which the field offset drops off considerably towards the top of the spinal cord (Fig. 2A). For spinal cord levels below C7, the field offset shows a tendency to level off or decrease again, though in most subjects the acquisition did not extend below the center of T1. The average (range) of the field offset over all subjects was 96 (46–211) Hz at the maximum offset near C7-T1, and 14 (6–25) Hz at C1. The skewed Gaussian model suggested by Verma et al., provided a good fit of the average and the individual field offsets (Fig. 2A and B). The resulting fit parameters are provided in the Supplementary Table.

### FLASH acquisitions

**Fig. 3:**
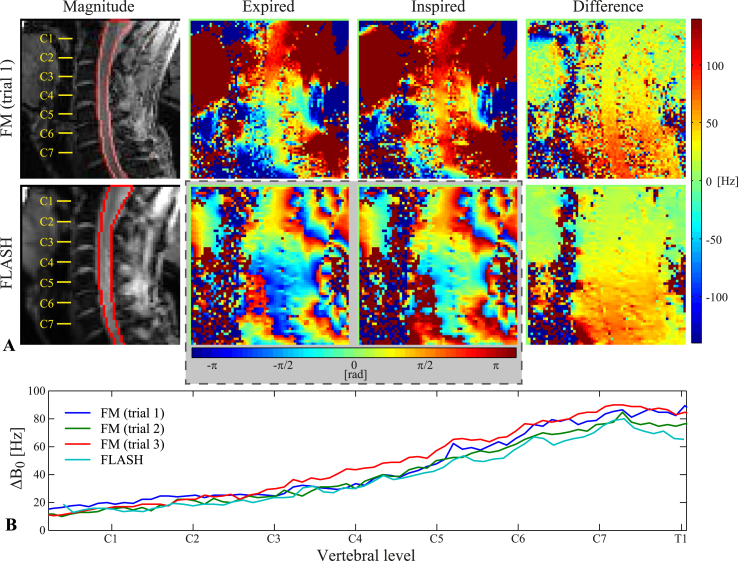
Comparison of field map and FLASH acquisitions during expired/inspired breath-holds. A) Top row: Field map acquisition, displaying the magnitude image with an outline of the spinal cord mask (left), the expired/inspired field maps (center) and the difference between the field maps (right). Bottom row: FLASH acquisition, displaying the mean magnitude image with an outline of the spinal cord mask (left), phase images at selected time points during expired/inspired breath-holds (center) and the difference between the phase images (right). B) The measured $\text{Δ}B_{0}\left(z\right)$ inside the spinal cord, shown for all three breath-hold field map trials and the FLASH acquisition at the selected time points shown in A.

Fig. 3 shows a comparison of field map and FLASH images acquired during expired and inspired breath-holds in one subject. For the FLASH acquisition, the temporally unwrapped phase images are shown from two selected time points during the breath-holds, and the $\text{Δ}B_{0}\left(z\right)$ is calculated from the phase difference between the two time points. The field difference images closely resemble each other in terms of magnitude and spatial distribution of the field offset. The measured $\text{Δ}B_{0}\left(z\right)$ of the FLASH acquisition largely falls within the variability between trials of the field map acquisition, though it is slightly lower in some locations (Fig. 3B).

**Fig. 4:**
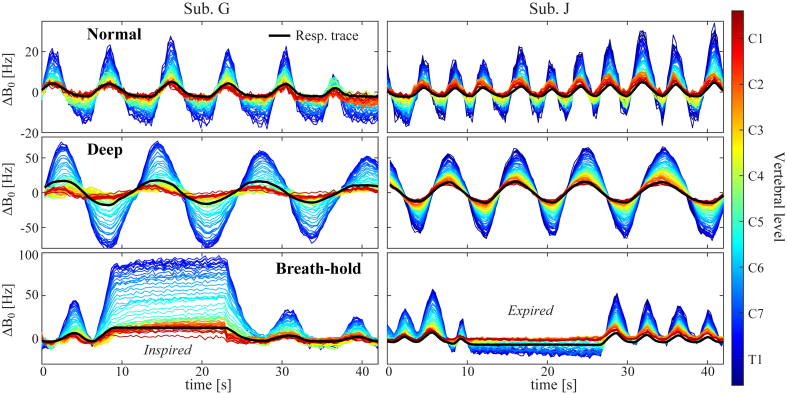
Time-courses of breathing-induced field fluctuations as measured by FLASH acquisitions during normal breathing (top), deep breathing (middle) and inspired/expired breath-holds (bottom) in two subjects. The line color indicates vertebral level. Overlaid in black is the simultaneously acquired trace from a respiratory bellow. Note the different scaling of the y-axis between the different breathing patterns.

The FLASH acquisition enabled time-resolved measurements of field fluctuations during free breathing, as demonstrated in Fig. 4. The time-courses of the measured field variations closely follow the respiratory trace. In these subjects, the trace was acquired without high-pass filtering. Peak-to-peak field fluctuations varied considerably between the different breathing conditions, with lower amplitudes during normal breathing as compared to deep breathing or breath-holds.

### PCA

**Fig. 5:**
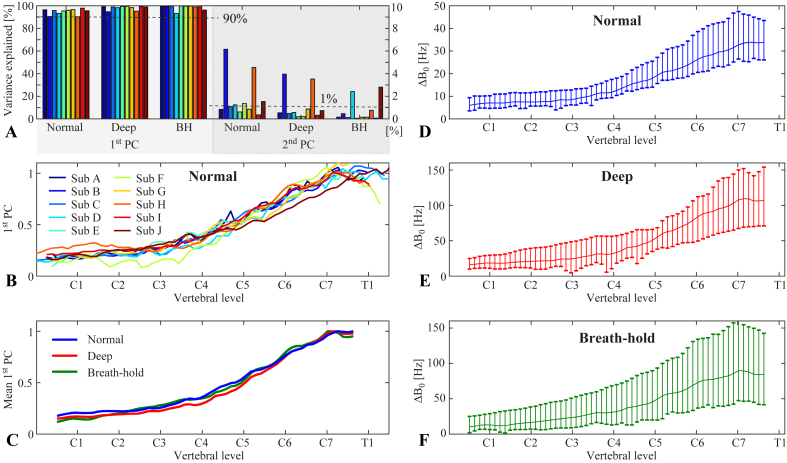
A) Percent variance explained by the first and the second principal component for all subjects and breathing conditions. Note the different scaling of the y-axis for the first and the second PC. B) Normalized first principal component of normal breathing for all subjects. C) Average of the first principal component over all subjects for normal breathing, deep breathing and breath-holds. D-F) Magnitude of field fluctuations as measured by the first principal component scaled by the difference between maximum and minimum values of the corresponding projection time-course. The plots show the mean (solid line) and the range (vertical bars) over all subjects, for normal breathing (D), deep breathing (E) and breath-holds (F).

Fig. 5 shows results of the principal component analysis on the field time-courses measured with the FLASH acquisition. The first principal component explained more than 90% of the field variance along the z-direction inside the spinal cord in all subjects and breathing patterns, whereas the second PC explained 1% or less in most cases (Fig. 5A). The z spatial profile of the first PC during normal breathing showed a high degree of similarity between subjects, though with some individual variations (Fig. 5B). The mean of the first PC over subjects was near identical between the different breathing states (Fig. 5C). Multiplying the first PC with the difference between the maximum and the minimum of its projection time-course yielded an estimate of the magnitude of peak-to-peak field fluctuations for each subject. The maximum field variation, around C7-T1, for normal, deep breathing and breath-holds was on average (range) 36 (27–47) Hz, 113 (72–154) Hz and 92 (49–165) Hz, respectively, whereas at the level of C1 it was 7 (5–10) Hz, 19 (11–29) Hz and 13 (6–29) Hz, respectively (Fig. 5D–F). The deep breathing thus generally induced 2–3 times larger field variations than normal breathing. The breath-holds often resembled deep breathing in magnitude, but had a higher degree of variability between subjects (Fig. 5F).

**Supp. Fig. 1:**
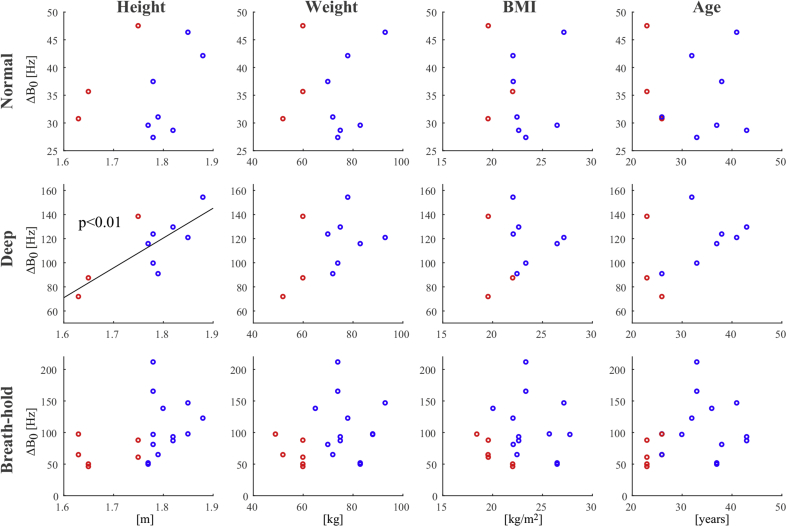
Maximum $\text{Δ}B_{0}\left(z\right)$ for the different breathing conditions, plotted against physical parameters of the different subjects. The female subjects are represented by red circles, and the male by blue circles. The breath-hold plots include both the field map and the FLASH acquisitions. The deep breathing showed a statistically significant (p < 0.01) correlation with subject height (r^2^ = 0.59).

Scatter plots of the maximum induced field shift vs. the subject's height, weight, BMI and age are shown in Supplementary Fig. 1. A trend towards higher field shifts with increasing height, weight, BMI and age was observed, especially in the case of deep breathing. The correlation was statistically significant only in the case of deep breathing vs. subject height (r^2^ = 0.59, p < 0.01).

### Field prediction

**Fig. 6:**
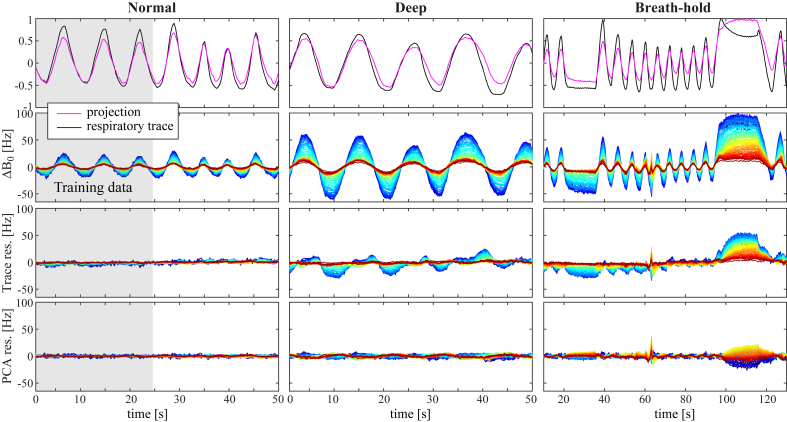
Results of modeling the field fluctuations based on the unfiltered respiratory trace or the PCA, demonstrated in one subject (Sub. I). The gray area indicates the training dataset. Top row: Time-courses of the respiratory trace and the projection of the first PC. Second row: The measured $\text{Δ}B_{0}\left(z,t\right)$. Third row: Residual after subtracting the contribution of the respiratory trace model. Bottom row: Residual after subtracting the contribution of the first PC. The color scale of $\text{Δ}B_{0}\left(z,t\right)$ is as in Fig. 4.

Fig. 6 displays results for one subject of predicting the field variations over time based on the unfiltered respiratory trace or on the first PC, using 30 s of normal breathing as training data. In the case of normal breathing, both the respiratory trace and the first PC explained most of the measured field variance. The first PC was also able to capture most of the field variation during deep breathing. It is interesting to note that the residual in this case showed similar amplitude at upper and lower levels of the spinal cord, but phase shifted relative each other. The residual of the PC model was somewhat larger during breath-holds. The respiratory trace, was less good as a predictor of the field during deep breathing and breath-holds, and the residual was considerably larger in these cases.

**Supp. Fig. 2:**
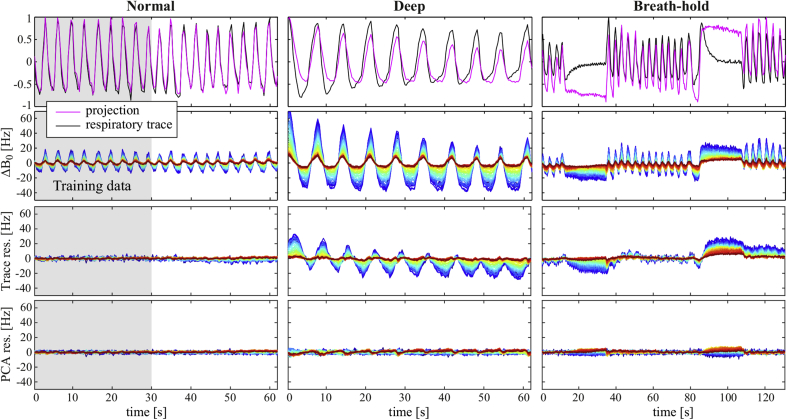
Results of modeling the field fluctuations based on the high-pass filtered respiratory trace or the PCA, demonstrated in one subject (Sub. A). The gray area indicates the training dataset. Top row: Time-courses of the respiratory trace and the projection of the first PC. Second row: The measured $\text{Δ}B_{0}\left(z,t\right)$. Third row: Residual after subtracting the contribution of the respiratory trace model. Bottom row: Residual after subtracting the contribution of the first PC. The color scale of $\text{Δ}B_{0}\left(z,t\right)$ is as in Fig. 4.

In Supplementary Fig. 2, the same analysis is shown for a subject where the respiratory trace was high-pass filtered. Also in this case, the normal breathing is well captured by the respiratory trace model. Deep breathing is partly captured, but with a large residual, whereas the breath-holds show almost no improvement in field variance by the trace model.

**Fig. 7:**
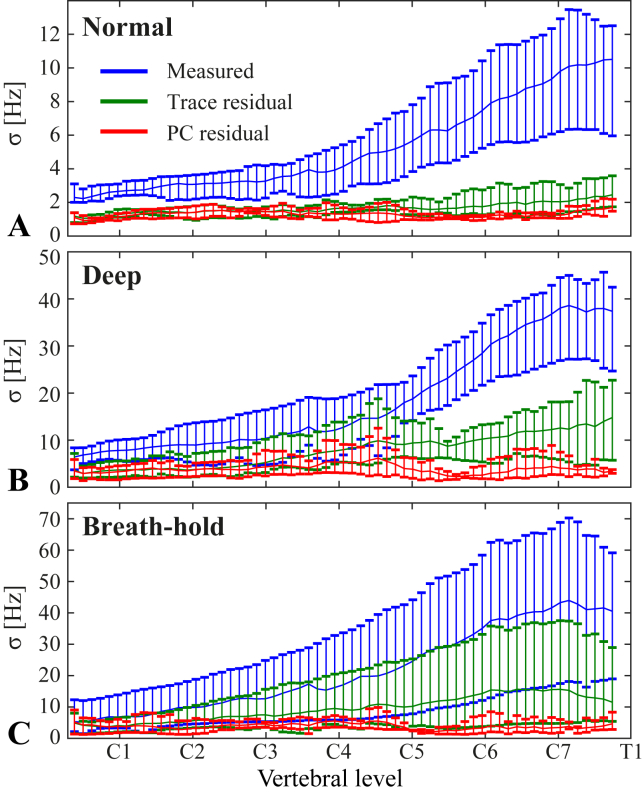
Standard deviation (σ) of the measured field fluctuations during normal breathing (A), deep breathing (B) and breath-holds (C), shown together with the standard deviation of the residual after subtracting the contribution of the respiratory trace model or the first principal component. The plots show the mean (solid line) and range (vertical bars) of the standard deviation over all subjects. Only subjects with an unfiltered respiratory trace (Sub. G-J) were included here.

Fig. 7 shows the standard deviation of the measured field time-courses and of the residual field after subtracting the component explained by the first PC or the respiratory trace model. Only data from the four subjects for which an unfiltered respiratory trace was acquired are shown here. In these subjects, the residual standard deviation at C7 during normal breathing was on average reduced from 10 Hz to 1 Hz by the PC model, and 2 Hz by the respiratory trace model. During deep breathing the PC model reduced the residual standard deviation at C7 from 38 Hz to 4 Hz, whereas the respiratory trace yielded a residual standard deviation of 13 Hz. Similar results as for deep breathing were seen for the breath-holds. Over all vertebral levels, the PC model yielded a residual standard deviation of on average 1–2 Hz for normal breathing, 2–6 Hz for deep breathing and 2–6 Hz for breath-holds, while the trace model yielded 1–2 Hz for normal breathing, 3–15 Hz for deep breathing and 4–16 Hz for breath-holds.

### In-plane PCA

**Fig. 8:**
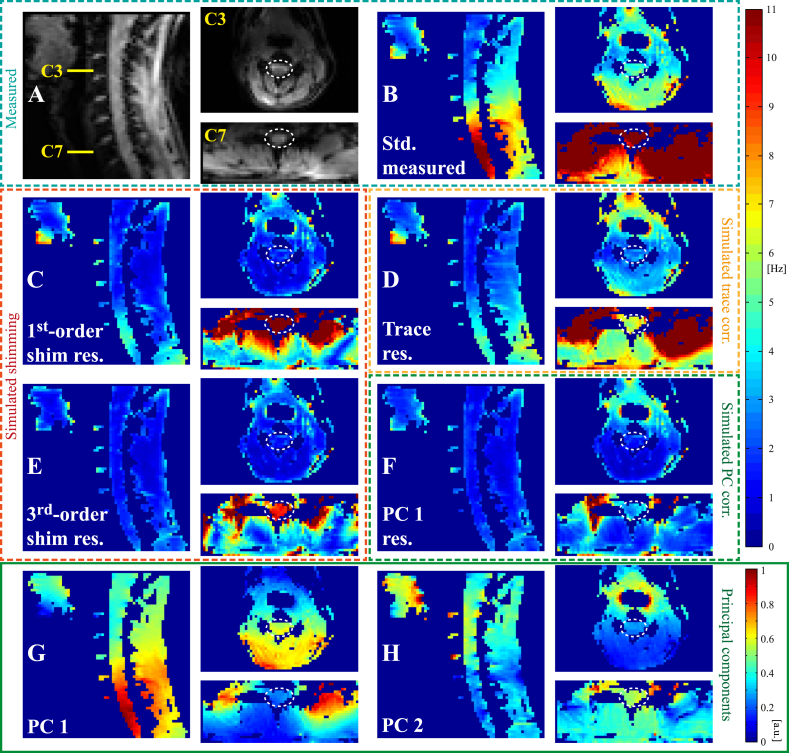
In-plane spatial distribution of breathing-induced field fluctuations in sagittal and transversal slices acquired during normal free breathing. A) Mean magnitude image of FLASH acquisition. B) Standard deviation of measured field fluctuations. D) Standard deviation of field residual after removing the field accounted for by the respiratory trace model (Simulated trace corr.). C,E) Standard deviation after subtracting the contribution of up to 1^st^- or 3^rd^-order spherical harmonic fields in-plane (Simulated shimming). F) Standard deviation of field residual after subtracting the contribution of the first in-plane PC (Simulated PC corr.). G-H) First and second in-plane principal component.

The analysis of the in-plane spatial distribution of the breathing-induced field fluctuations is demonstrated for one subject in Fig. 8. Fig. 8A and B shows the mean magnitude image and the standard deviation of the field over time, respectively, of a sagittal slice and two transversal slices at the vertebral levels of C3 and C7. A simulated slice-wise B_0_ field correction based on the respiratory trace reduced the standard deviation of the field in the spinal cord at all levels (Fig. 8D). However, as the correction assumed a uniform field offset in the transverse plane, considerable levels of field fluctuations remained in surrounding tissue, especially at the level of C7.

Simulated shim correction of up to 1st order removed most of the field variations in the transversal slice at the level of C3 (Fig. 8C). Also in the sagittal plane, 1st-order fields provided the largest contribution to the correction, with gradual further improvement up to 3rd order (Fig. 8C,E). In the transversal plane at C7, considerable field variance remained within the slice also after 3rd-order field correction (Fig. 8E).

At the level of C3, the first in-plane PC largely consisted of an anterior-posterior field gradient (Fig. 8G). Interestingly, the second PC showed a smaller field component centered on the airways (Fig. 8H). This component may be caused by small volume changes of the airways, or a difference in oxygen fraction between inspired and expired air. Removing the contribution of the first PC from the measured field at this level yielded lower residual standard deviation than with the respiratory trace model, but higher than with simulated shimming, indicating that the second component provided a significant contribution (Fig. 8F). At the level of C7 the spatial field distribution of the first PC was more complex than higher up in the neck, with hot spots above the tip of the lungs on either side (Fig. 8G). Here, the residual after removing the contribution of the first PC was lower than achieved with up to 3rd order shim fields (Fig. 8F).

### Anatomical multi-echo GRE

**Fig. 9:**
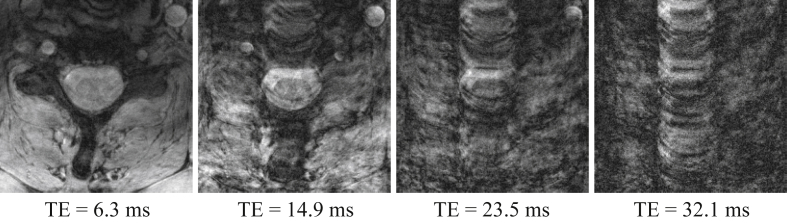
Magnitude images of every second echo of the high-resolution GRE acquisition in one slice at the vertebral level of C6.

The high-resolution structural GRE acquisition showed increasing levels of ghosting with increasing echo time (Fig. 9). The first echo at 6.3 ms shows only subtle intensity variations over the image, whereas the last echoes are completely corrupted by the ghosting. Note that all echoes were obtained in the same acquisition and thus were exposed to the same field perturbations. The dependence on echo time indicates that the source of the ghosting is due to field variations, as field offsets cause phase errors to accumulate with time after excitation. Actual motion could cause similar ghosting in single images, but would be affecting all echoes equally.

### Shim correction

**Fig. 10:**
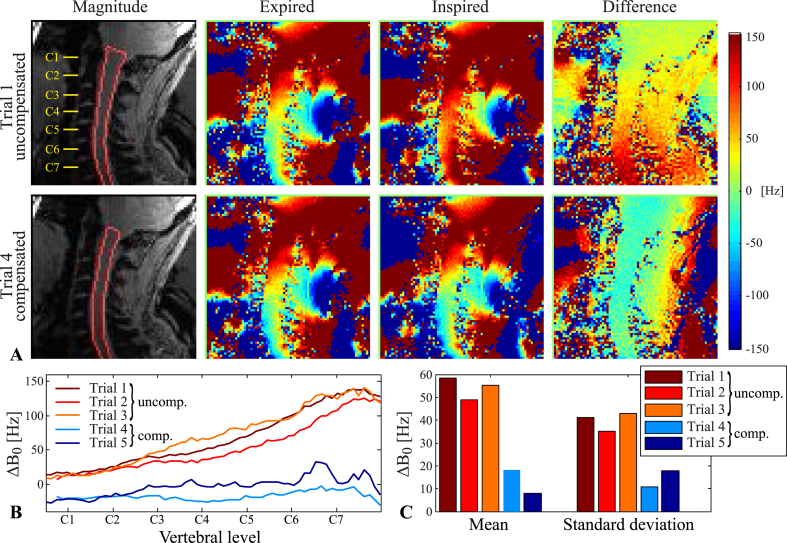
Proof-of-principle shim compensation of the breathing-induced fields in one subject. A) Top row: Field maps acquired during expired/inspired breath-holds, used to calculate the 2^nd^-order shim compensation settings. Bottom row: Field maps acquired during expired/inspired breath-holds, with compensation shims applied during the inspired breath-hold. B) The measured $\text{Δ}B_{0}\left(z\right)$ inside the spinal cord in three trials without shim compensation (1–3, uncomp.) and two trials with shim compensation (4–5, comp.). C) Mean and standard deviation of the measured breathing-induced field offset over all voxels inside the spinal cord mask.

Fig. 10 shows a proof-of-principle shim compensation during breath-hold field map acquisitions in one subject. The field difference between inspiration and expiration was considerably reduced by the 2nd-order shim compensation. With compensation, $\text{Δ}B_{0}\left(z\right)$ maximally peaked at around 30 Hz, compared to 140 Hz without compensation, and the variance along the cord was less (Fig. 10B). The mean and the standard deviation of $\text{Δ}B_{0}$ over all voxels within the spinal cord mask were reduced by a factor of 2–3 as compared to uncompensated acquisitions. Remaining offsets are likely to be attributed to variations in the breath-holds between trials, but may also be partly due to incomplete calibration of the actual shim field profiles of the second-order shims.

### Swallowing

**Fig. 11:**
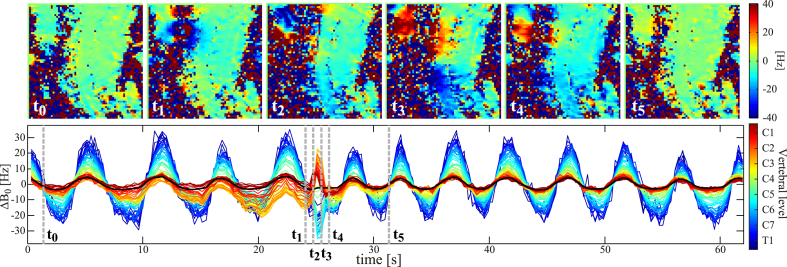
FLASH acquisition capturing one swallowing event. The top row shows the measured field at time points before, during and after the swallow, and the bottom row shows the resulting $\text{Δ}B_{0}\left(z\right)$ for the full acquisition. Dashed vertical lines indicate the time points of the images in the top row.

Occasionally, subjects swallowed during the free breathing acquisitions, thereby incidentally yielding a recording of the field changes associated with swallowing. One such event is presented in Fig. 11, revealing a substantial influence on the field distribution down to about the vertebral level of C7. In the middle of the swallow, the field increased by up to 20 Hz in the upper cervical spine (C2-C4), while decreasing by about 30 Hz in the lower cervical spine (C5-C7). A field drift over 10–20 s can be noticed in the upper cervical spine in the time leading up to the swallow, presumably due to tissue shifting slowly in and around the mouth. A video of the swallowing event and adjacent breathing-induced field fluctuations is shown in the Supplementary Video.

Supplementary video related to this article can be found at https://doi.org/10.1016/j.neuroimage.2017.11.031

The following is the supplementary data related to this article:

Supp. Video Video of FLASH magnitude and field images acquired during normal free breathing in one subject. The acquisition incidentally captured one swallowing event. The corresponding $\text{Δ}B_{0}\left(z\right)$ is shown beneath the images, and the current time point is indicated by the dashed vertical line. Supp. Video

**Supp. Fig. 3:**
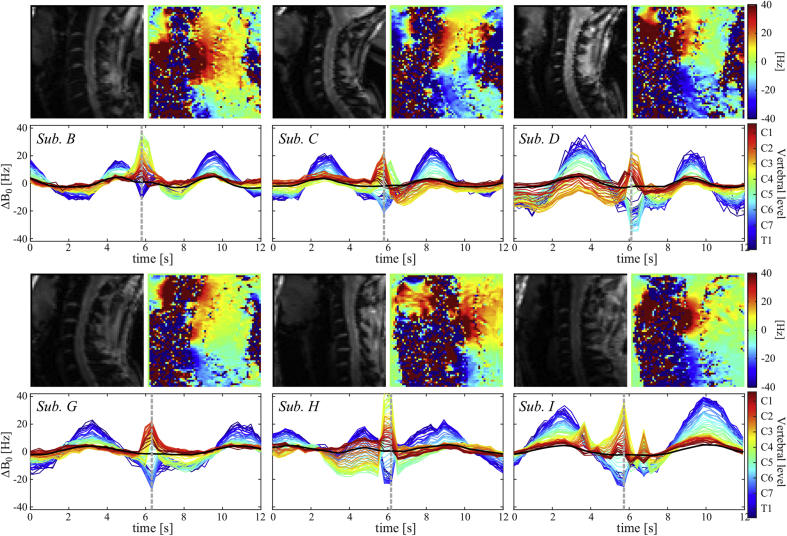
Field images and time-courses of swallowing for all six subjects in whom a swallowing event was captured during the FLASH acquisitions. The magnitude images are from the start of the acquisition and are displayed for spatial orientation.

The field shift induced by swallowing showed a similar spatial field distribution, and a peak magnitude of about 20–40 Hz, in a number of different subjects. This is demonstrated in Supplementary Fig. 3, for all six subjects who swallowed at some point during the FLASH acquisitions. None of the subjects was instructed to swallow.

## Discussion

### $\Delta B_{0}$ magnitude and profile

Breathing is associated with chest motion and changing lung volume, which changes the B_0_ field distribution also at a distance from the thorax. Here, we measured breathing-induced B_0_ fields in the cervical spinal cord at 7T, observing a peak-to-peak field shift around C7 of on average 36 Hz during normal free breathing. During deep breathing, this increased to 113 Hz on average, indicating that the breathing pattern strongly influences the magnitude of the field fluctuations. In all subjects, the field shift was largest around the transition between the cervical and the thoracic spine, and lowest at the top of the spinal cord. At the level of C1, the observed field shift was on average 7 Hz during normal breathing, which is broadly consistent with previous 7T studies reporting breathing-induced field shifts on the order of a few hertz in the brain and brainstem (Duerst et al., 2016, Van de Moortele et al., 2002, van Gelderen et al., 2007).

The spatial profile of the measured breathing-induced fields in the spinal cord was consistent with results from a previous study at 3T (Verma and Cohen-Adad, 2014), and was well described by the empirical mathematical model suggested in that study. The same 3T study reported an average field shift of 74 Hz at the level of C7 between inspired and expired breath-holds. The field shift observed during breath-holds in our data was on average around 90 Hz, which is lower than expected by directly scaling the 3T results to 7T. The reasons for this discrepancy are not clear. Here, we observed that breath-holds resembled deep breathing in magnitude, but with a larger variability between subjects. This variability may reflect differences in depth of expiration/inspiration during breath-holds, on top of the inter-individual anatomical differences. A systematic difference between the studies in the depth of breath-holds could thus potentially underlie the observed field difference. Another relevant factor may be the background B_0_ field profile off from isocenter of the different MR systems, as it determines the magnetization of tissue in the thorax and abdomen.

Factors such as age, height and weight may influence the magnitude or spatial distribution of breathing-induced field fluctuations. In the brain, a relation between the magnitude of breathing-induced fields and the BMI of the subject has been noted (Duerst et al., 2016). Here we observed a statistically significant correlation between subject height and maximum induced field shift during deep breathing, and there appeared to be a non-significant trend also for weight, BMI and age. This is in agreement with trends between breathing-induced field in the spine and height/weight previously observed at 3T (Verma and Cohen-Adad, 2014). The current sample size was however relatively small, and not fully representative of the variability in physical parameters present in the general population. Further investigation would therefore be needed to validate the results.

The character of breathing, for example the relative usage of the diaphragm or accessory muscles for respiration, may also influence the resulting field shifts. Preliminary observations in a single subject (data not shown) suggest that the amplitude of breathing-induced fields in the cervical spine may be higher if the subject is breathing more with the chest. This observation however remains to be confirmed through further investigation.

### Resulting image artifacts

The impact of field fluctuations on image quality depends on the type of sequence used. Gradient echo sequences with long echo times and/or long readouts are especially vulnerable, as the field offset causes phase to accumulate linearly over time after excitation. In multi-shot acquisitions, this leads to phase inconsistencies between k-space lines, which can give rise to ghosting and blurring, as well as ringing and signal intensity modulations over the image. We here demonstrated the severity of ghosting that can occur in high-resolution T2*-weighted images of the spine during normal breathing, when no correction is applied. The level of ghosting increased with the echo time of the acquisition, indicating B_0_ field variations rather than actual motion as the source. At echo times of 20 ms and above, the images were completely corrupted by ghosting. Structural T2*-weighted acquisitions of the spinal cord at 7T hold great promise for their excellent gray/white matter contrast (Sigmund et al., 2012, Zhao et al., 2014) and sensitivity to detect pathologies (Dula et al., 2016), and effective correction strategies will therefore be crucial.

In single-shot acquisitions, the field fluctuations primarily cause data inconsistencies between images in a time-series. For single-shot EPI, this predominantly manifests as apparent motion between images, but the field fluctuations can also contribute to variable ghosting and distortion. Assuming a phase encoding bandwidth of 15 Hz/pixel and a resolution of 1 mm, the magnitude of field fluctuations measured here imply apparent motion of about 2 mm during normal breathing and 7 mm during deep breathing. Considering the small cross-section of the spinal cord, this will severely affect any time-series analyses if not corrected for. Single-shot EPI is the sequence most commonly used to measure blood-oxygen level dependent contrast in functional MRI. Model-based and data-driven removal of physiological confounds is therefore a crucial component of the data processing in fMRI of the spinal cord (Barry et al., 2014, Barry et al., 2016, Brooks et al., 2008, Kong et al., 2012). Incomplete removal of physiological noise can reduce the sensitivity to detect functional activation, as well as increase the rate of false positives. A further complication for fMRI of the spinal cord at 7T is the short T2* due to macroscopic static field inhomogeneity, which causes rapid signal decay. To reduce the echo time, and thereby reduce signal loss due to de-phasing, spinal cord fMRI at ultra-high field has so far been performed with 3D segmented EPI acquisitions (Barry et al., 2014, Barry et al., 2016). Segmented EPI is however more sensitive to ghosting due to phase inconsistencies between shots, as observed here in the anatomical acquisitions.

### Correction strategies

We here demonstrated 2nd order shim compensation of breathing-induced fields during breath-holds as a proof-of-principle correction. Ideally, a correction method should be able to address field fluctuations during free breathing. This requires two components: i) accurate temporal tracking of the state of the field, and ii) dynamic compensation for the estimated field fluctuations.

The results presented here suggest that a single tracker representing the first principal component of the breathing state would be able to predict a large part of the field variance along the foot-head axis inside the spinal cord. One approach is to let the respiratory trace serve as indicator of the breathing state, using training data to translate it into field (van Gelderen et al., 2007). With the setup used in this study, such an approach seems feasible for normal breathing, but would be less accurate for deep or irregular breathing. The accuracy of the field tracking can be affected by the specific setup used for the respiratory trace (type of respiratory bellows, pressure sensor, signal processing, exact placement of bellows), and it may thus be possible that further optimization would yield a more reliable trace also for deep breathing. Even so, it is not necessarily given that complete linearity between thorax/abdominal circumference, as measured by the respiratory bellows, and the field state exists. For practical purposes, however, the linearity appears to be sufficient during normal, shallow breathing using a standard respiratory trace.

A further approach to track the field is to use external field sensors placed in the vicinity of the subject, combined with a model to infer the field inside the imaging volume. The use of several sensors yields the possibility of tracking different temporal components, which is beneficial in cases where more than one principal component provides a significant contribution to the field variations. In brain imaging, NMR (Nuclear Magnetic Resonance) field probes have been deployed for this purpose, using a model based on spherical harmonic expansion (Duerst et al., 2015, Vannesjo et al., 2015) or on training data (Wezel et al., 2017). Given the measured spatial structure of breathing-induced fields in this work, a spherical harmonic model may be applicable in upper parts of the cervical spine, whereas lower regions would likely require training-based or prior information in the model.

As an alternative to external trackers, navigators can be used to directly measure field offsets in tissue. This is straightforward for uniform fields (Versluis et al., 2010) and field gradients in one direction, as observed in the upper part of the neck, but requires more sophisticated strategies to measure complex 2D or 3D spatial field distribution (Versluis et al., 2012), as present closer to the thorax. The navigator approach can capture field perturbations of various sources, such as swallowing and system field drifts, and does not require assumptions about temporal stationarity (i.e. a fixed relationship between physiological state and the induced field). This may be particularly advantageous for subjects who are breathing irregularly or swallowing frequently, as the induced field fluctuations will be harder to capture with external trackers in this case.

Provided that accurate tracking of the field fluctuations can be obtained, the next step is to compensate for their effects. One approach is to retrospectively apply corrections at the level of image reconstruction. Such an approach is feasible when the field perturbations are sufficiently small, such that the reconstruction problem remains well posed. For uniform field offsets, retrospective data correction by signal demodulation is straightforward. Such correction implemented on a slice-by-slice basis in transversal acquisitions would eliminate most breathing-induced field fluctuations inside the spinal cord itself. Non-uniform field fluctuations, as present in the full image plane, can be incorporated in the encoding model of the image reconstruction (Vannesjo et al., 2015, Versluis et al., 2012). First-order fields, as observed in the upper parts of the neck, can be accommodated by the common k-space framework, while fields of arbitrary spatial structure can be included in a general encoding model (Wilm et al., 2011).

Alternatively, a prospective correction approach can be employed, using the shim system for real-time field stabilization (Duerst et al., 2015, Topfer et al., 2017, van Gelderen et al., 2007). On most systems, this requires a dedicated hardware setup to control the higher-order shim channels in real-time. Prospective field compensation has the advantage that effects on for example slice excitation and signal de-phasing can be actively counteracted. However, compensation field profiles are limited by the available shim system, which commonly produces spherical harmonic fields of up to 2nd or 3rd order. The data acquired here indicate that up to 3^rd^-order spherical harmonic fields can approximate the breathing-induced fields well inside the cervical spinal cord itself. In surrounding tissue, first-order fields can describe most of the field variations in the upper parts of the neck. Closer to the thoracic spine, however, the transversal in-plane field distribution is more complex and cannot be fully described by spherical harmonic terms of low order. Shim coils specifically designed for the spinal cord may provide more degrees of freedom to approximate the field profiles induced by breathing. A 24-channel spinal cord shim coil has been described for this purpose (Topfer et al., 2016), and has been used for prospective field correction based on signal from a respiratory trace in EPI acquisitions of the spinal cord at 3T (Topfer et al., 2017).

### Swallowing and motion

This work has focused on B_0_ field fluctuations caused by breathing as a major source of physiological noise in ultra-high field spinal cord imaging. There are however a number of other physiological effects that can influence data quality. As observed here, swallowing is both associated with tissue motion, as well as field changes of up to around 40 Hz, affecting the full length of the cervical spinal cord. Swallowing and speaking have previously been observed to induce field changes in the brain (Birn et al., 1998). Cardiac pulsation leads to pulsatile flow of the cerebrospinal fluid surrounding the spinal cord (Greitz et al., 1993). Respiration causes slight motion of the head and neck, and further down in the thoracic and lumbar spine, the chest motion of breathing will be within the field of view of the acquisitions. Subject motion unrelated to breathing may also be present during acquisitions, especially in a general patient population. This may affect image quality through motion of the imaging volume itself, as well as through motion-induced B_0_ field changes.

As the demands on resolution and accuracy of spinal cord imaging increase, so will the need for minimizing physiological influences on the data. Different approaches may be required to address the various sources of physiological noise. Targeting breathing-induced field fluctuations is an initial step to remove a dominant source of physiological noise present in all subjects.

## Conclusions

In this work, we have measured breathing-induced field fluctuations in the cervical spinal cord at 7T. Field shifts of on average 36 Hz during normal free breathing, and 113 Hz during deep breathing were observed in the lower cervical spine. For comparison, respiratory field fluctuations on the order of a few hertz have been reported to cause substantial artifacts in high-resolution T2*-weighted brain imaging. To unlock the full potential of ultra-high field for spinal cord imaging, it will hence be crucial to address the dynamic B_0_ fields in free breathing acquisitions. A proof-of-principle correction using the 2nd order shim system was demonstrated during breath-holds. To extend this to free-breathing acquisitions it will be necessary to track the breathing state in real-time. As more than 90% of the field variance inside the spinal cord could be explained by a single principal component, one or a few sensors, e.g. a respiratory trace or external field probes, may be sufficient for this purpose.

## Appendix A Supplementary data

Supp. Table Fit parameters of the skewed Gaussian model. Supp. Table
