# Supplementary material for: Spatiotemporal characterization of breathing-induced B0 field fluctuations in the cervical spinal cord at 7T
Source: Neuroimage. 2018 Feb 15;167:191–202. doi: 10.1016/j.neuroimage.2017.11.031 (PMC5854299; doi:10.1016/j.neuroimage.2017.11.031)
Supplement: Supp. Table — Fit parameters of the skewed Gaussian model. [file mmc1.docx]

|  | *a* | *μ* | *σ* | *s* | *b* | *c* |
| --- | --- | --- | --- | --- | --- | --- |
| Mean | 119 | 1.15 | 0.39 | -9.30 | -0.0 | 11.2 |
| s1 | 73 | 0.95 | 0.29 | -0.07 | -0.0 | 7.3 |
| s2 | 55 | 1.13 | 0.31 | -4.83 | 31.6 | 13.8 |
| s3 | 173 | 1.20 | 0.49 | -7.07 | -0.0 | 1.3 |
| s4 | 210 | 1.26 | 0.48 | -7.32 | -0.0 | 9.6 |
| s5 | 57 | 1.06 | 0.31 | -11.53 | 30.8 | 17.0 |
| s6 | 110 | 1.14 | 0.32 | -4.98 | 12.8 | 5.8 |
| s7 | 54 | 1.22 | 0.36 | -7.57 | -0.0 | 5.9 |
| s8 | 285 | 1.16 | 0.36 | -5.76 | -0.0 | 20.0 |
| s9 | 220 | 0.97 | 0.34 | 0.36 | -70.3 | 18.3 |
